# Genome‐Wide Association Study Reveals Insect Genetics and Microbial Symbiont Effects on Susceptibility of *Diaphorina citri* to the Citrus Greening Pathogen, *Candidatus* Liberibacter Asiaticus

**DOI:** 10.1002/advs.202517056

**Published:** 2026-03-10

**Authors:** Kai Liu, Qingcui He, Zeyue Lin, Shixuan Huang, Zichun Zhong, Pingyang Zhu, Mengge Gao, Luyao Zhao, Han Jin, Guiting Wu, Gurr M. Geoff, Qunxin Han, Rui Pang

**Affiliations:** ^1^ Key Laboratory of Green Prevention and Control on Fruits and Vegetables in South China Ministry of Agriculture and Rural Affairs College of Agriculture and Biology Zhongkai University of Agriculture and Engineering Guangzhou Guangdong P. R. China; ^2^ State Key Laboratory of Green Pesticide College of Plant Protection South China Agricultural University Guangzhou Guangdong P. R. China; ^3^ College of Life Sciences Zhejiang Normal University Jinhua P. R. China; ^4^ Gulbali Institute Charles Sturt University Orange New South Wales Australia

**Keywords:** citrus greening pathogen, diaphorina citri, genetics, susceptibility, symbiotic microbiota

## Abstract

Insect‐vectored pathogens pose a significant threat to global agriculture. The colonization efficiency of pathogens in vectors plays a central role in these pathosystems, yet studies of the factors that affect this aspect are limited. This study investigates the genetic and microbial symbiont factors influencing the susceptibility of *Diaphorina citri* to *Candidatus* Liberibacter asiaticus (*C*Las), the pathogen causing citrus greening disease (huanglongbing). Through a microbiome Genome Wide Association Study (mGWAS) based on 16S amplicon sequencing and genomic resequencing of 120 *D. citri* individuals from six populations, we identified 79 SNPs significantly associated with the relative abundance of *C*Las within insects. Additionally, some of these SNPs were also associated with the relative abundance of *Candidatus* Profftella armature, a key endosymbiont of *D. citri*. SNPs in the regulatory region of gene *Dcitr04g11610.1* led to its overexpression in *C*Las‐susceptible *D. citri*, and *C*Las infection further elevated its expression. Conversely, RNAi knockdown of *Dcitr04g11610.1* reduced *C*Las infection rates and abundance, accompanied by increased abundance of *Profftella*. Phylogenetic analysis revealed *Dcitr04g11610.1*’s high homology to Major Facilitator Superfamily‐type transporter SLC18B1 proteins, suggesting a role in *C*Las polyamine utilization. These findings highlight the importance and potential interplay of insect genetics and symbiotic microbiota in insect‐vectored plant pathogen systems.

## Graphical abstract

This study investigates the genetic and microbial factors influencing the susceptibility of *Diaphorina citri* to the citrus greening pathogen *Candidatus* Liberibacter asiaticus (*C*Las), employing a microbiome Genome Wide Association Study. The research identifies a key gene encoding an MFS‐type transporter contributing to *C*Las infectivity and abundance in *D.citri*, possibly by regulating the polyamine transport and endosymbiont *Profftella* abundance.

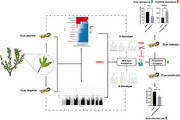

## 1 Introduction

Arthropods are common natural vectors for microbial pathogen transmission. Mosquitoes and ticks are notorious vectors for various human pathogens, including the dengue and Zika viruses, causing a public health threat worldwide [1, 2]. In agriculture, numerous plant pathogen groups, including bacteria, fungi, and approximately 80% of the currently known plant viruses, are transmitted through insect vectors [3]. Insect‐transmitted bacteria cause devastating disease outbreaks in over 300 major crop plant species, which greatly affects farmer incomes and poses a great threat to global food security [4, 5]. Therefore, it is important to identify the factors that can influence the capacity of vectors to acquire and transmit pathogens.

Huanglongbing (HLB), also known as citrus greening disease, represents the most devastating biotic threat to global citrus production, causing annual losses of billions of dollars and threatening the economic viability of citrus industries worldwide [6, 7]. This disease has spread invasively across Asia, Africa, and the Americas, where effective control remains challenging due to the lack of resistant commercial cultivars and limitations of current management strategies [8, 9]. The obligatory phloem‐limited bacterium *Candidatus* Liberibacter asiaticus (*C*Las) is the primary pathogen associated with HLB in these regions, and its transmission is exclusively mediated by insect vectors [10].

The Asian citrus psyllid, *Diaphorina citri* Kuwayama (Hemiptera: Liviidae), is the principal vector of *C*Las, and these two species have evolved an intimate relationship in which the pathogen colonises, reproduces, and persists within the host. Following acquisition by feeding on infected plants, *C*Las circulates within the psyllid, traversing gut barriers to ultimately colonize the salivary glands, a prerequisite for successful transmission [11]. The presence of infected psyllids enables rapid HLB transmission through citrus groves, resulting in substantial damage and considerable yield reduction. Importantly, however, substantial variation in *C*Las colonization efficiency exists among individual psyllids and field populations, with a subset of exposed insects more rapidly developing persistent infections or achieving transmission capability [12, 13, 14]. Understanding the determinants of this variable, *C*Las colonization efficiency, is critical for developing novel vector‐based disease control strategies.

Emerging evidence suggests that both microbial symbionts and host genetic variations may contribute to this phenomenon. In *D. citri*, the endosymbionts *Candidatus* Profftella armatura and *Wolbachia* species have been implicated in modulating *C*Las infection dynamics [15, 16]. More broadly, studies of disease vectors demonstrate that microbial symbionts can influence pathogen colonization through direct antagonism, immune priming, or nutrient competition [17, 18, 19, 20]. However, these effects are often context‐dependent and vary with host genotype. For example, the Q‐biotype *of Bemisia tabaci* is more susceptible to tomato yellow leaf virus infection than the B‐biotype [21]. Similarly, specific immune differences between genotypes have resulted in variations in susceptibility to Zika infections in *Aedes aegypti* adults [22]. Although intraspecific genetic variation has been documented to affect vector susceptibility in other systems, the specific host genetic factors governing *C*Las colonization in *D. citri* and their potential interaction with microbial symbionts remain uncharacterized.

To explore this issue, we collected *D. citri* samples from six different locations and with differing genetic backgrounds and tested for *C*Las colonization. Based on this, 16S amplicon sequencing and genomic resequencing were used to determine the microbial symbiont diversity and genetic variations in these samples. Finally, through microbiome‐genome wide association study (mGWAS), we aimed to identify the genetic factors and potential gene‐symbiont interactions that influence the susceptibility of *D. citri* to *C*Las.

## 2 Results

### 2.1 Microbial Composition of *D. citri* Populations

**FIGURE 1:**
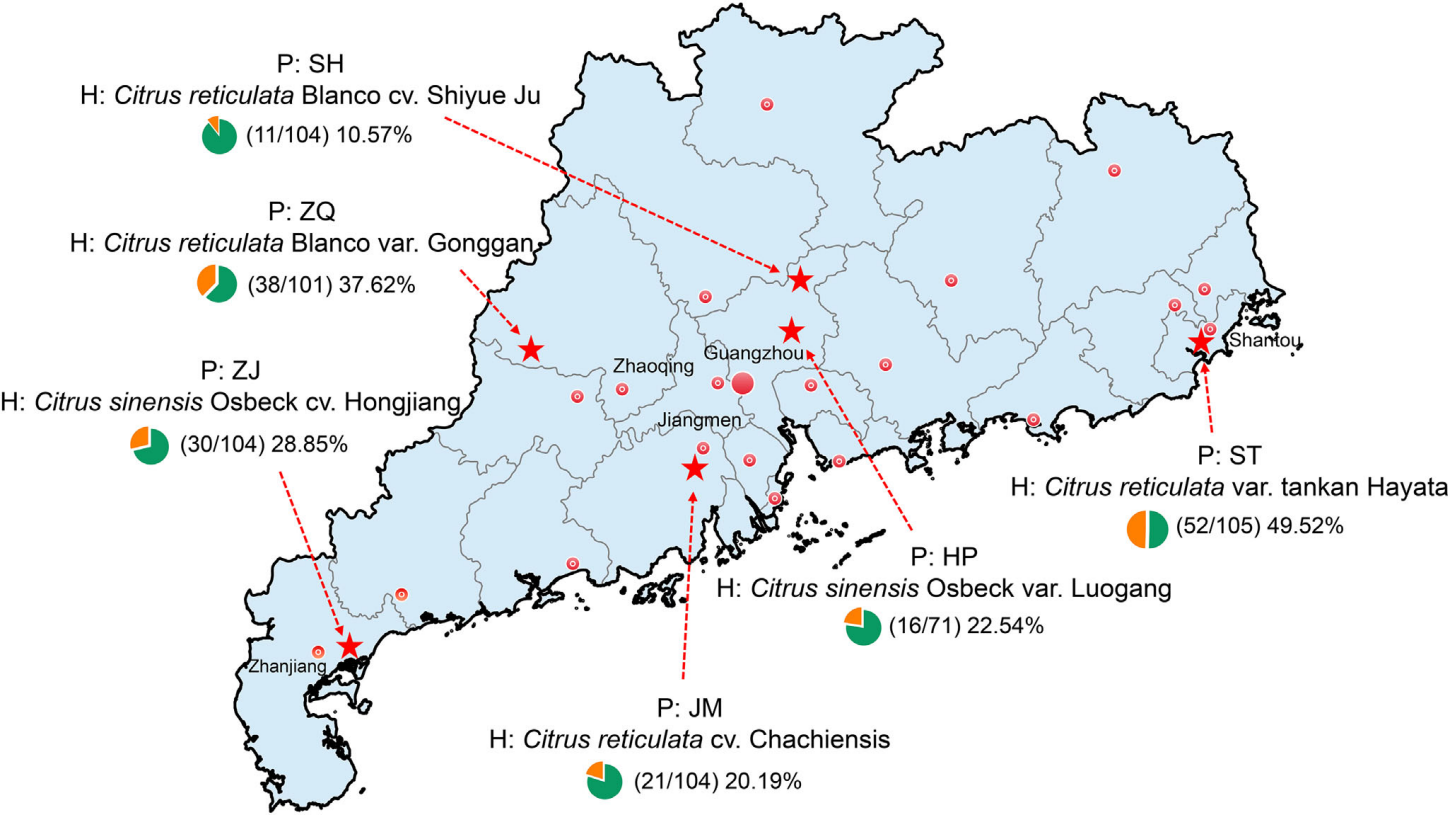
Sampling of *D.citri* in different geographical populations of Guangdong in southern China. P: population name; H: host plant from which the population in this region was recovered. Red stars on the map mark the sampling locations, and detailed latitude and longitude information is provided in Table S1. Pie charts indicate the infection rates of *Candidatus* Liberibacter asiaticus in samples collected from the corresponding region, detected using conventional PCR. Map of Guangdong province is drawn according to Natural Earth (https://www.naturalearthdata.com).

**FIGURE 2:**
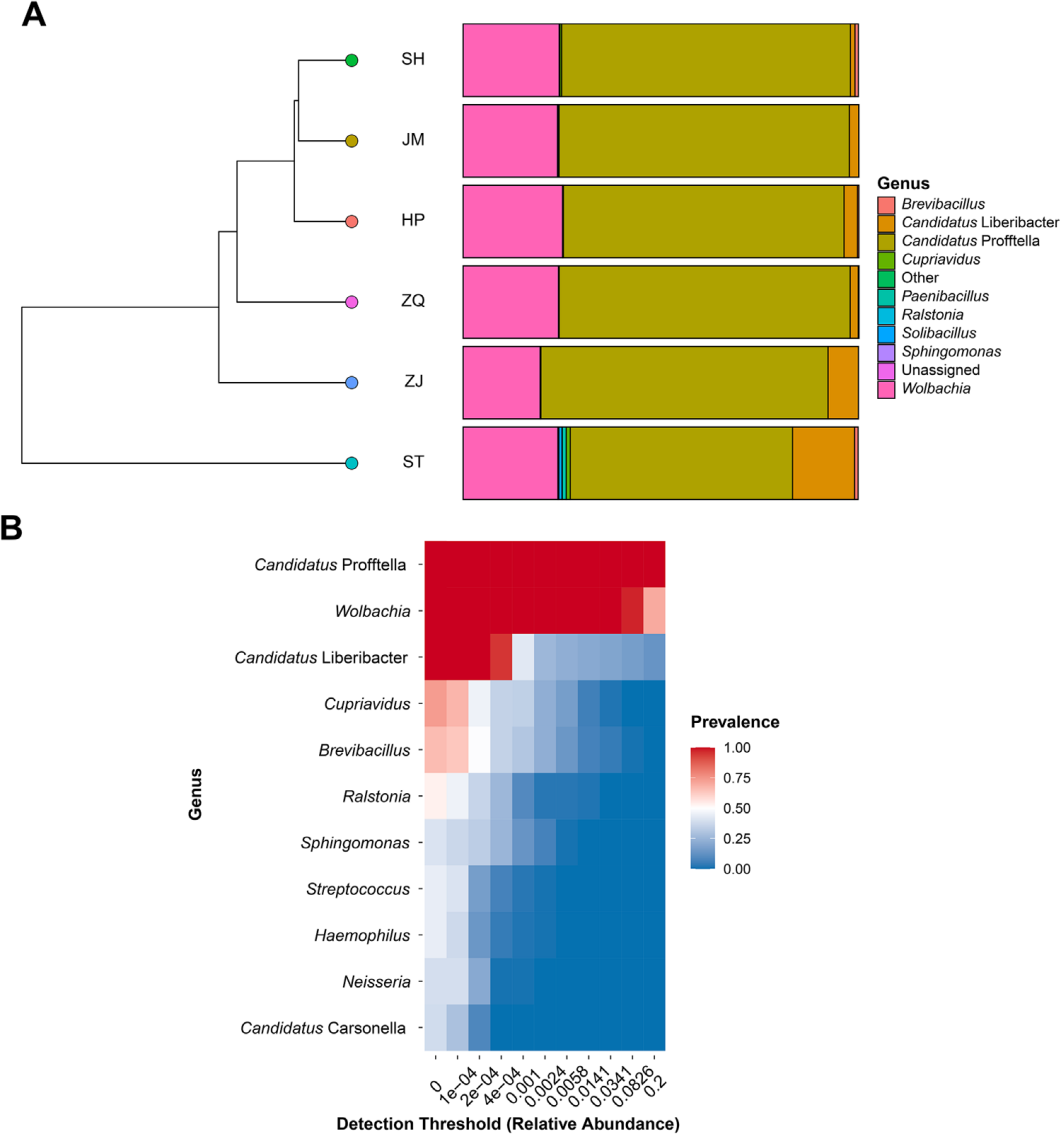
Microbial composition and core genera in different *D.citri* populations. (A) Relative abundance of bacterial communities at the genus level in each population. (B) Heatmap illustrating the prevalence of the major genera at different relative abundance thresholds among all samples.

To characterize the microbial composition of *D. citri*, we analyzed 16S rRNA sequencing data from a total of 120 samples from six geographic populations that were collected from different regions of Guangdong, a province in southern China where *D. citri* is native and HLB has occurred for a century. Analyses revealed highly variable *C*Las infection rates under conventional PCR detection (Figure 1; Table S1). After filtering, 11.57 of 14.73 million clean paired‐end reads were clustered into 264 amplicon sequence variants (ASVs) for all the samples (Tables S2 and S3). After the taxonomic classification of ASVs, we found that the dominant genera were *Candidatus* Profftella (average 69.83%) and *Wolbachia* (average 23.43 %) (Figure 2A; Table S4). Present also was *Candidatus* Liberibacter, for which relative abundance was highest in the Shantou (ST) population in the east of Guangdong (15.64%), followed by in the Zhanjiang (ZJ) population in the west (7.65%). Notably, the ASV representing *Candidatus* Liberibacter was found in all samples, including PCR‐negative samples (Figure S1). However, the presence of *C*Las in PCR detection showed a significantly positive correlation with the relative abundance of *C*Las in sequencing data (Pearson's correlation coefficient = 0.61, *p* = 2.01e^−13^). We assumed that the *C*Las abundance in *D. citri* sample in which *C*Las had successfully colonized was high enough to be definitively detected using conventional PCR, and PCR‐negative samples indicated that *C*Las had not colonized. Then we counted the relative abundance of *C*Las in PCR‐negative samples and used the upper limit of the 95% confidence interval (CI), 1.33%, as the threshold for determining *C*Las colonization in *D. citri*. Only the samples containing *C*Las abundance higher than this value were considered as colonized. The maximum difference between two continuous abundance values occurring at samples on either side of the threshold (1.84% for sample HP‐1 and 0.85% for sample ST‐11) supported our assumption (Figure S1).

We further defined the core microbial symbiont genera in *D. citri* according to the relative abundance and prevalence of each taxon. Only five genera were present in more than half of the samples under the threshold of relative abundance over 1e^−4^ (Figure 2B). When the relative abundance threshold was set to 1e^−3^, only two endosymbionts (*Candidatus* Profftella and *Wolbachia*) were retained, which was in accordance with the previous studies showing relatively low diversity and abundance of gut microbiome members in most Hemipteran insects [23, 24, 25].

### 2.2 Host Insect Genetic Loci Associated with the Relative Abundance of *C*Las

Microbiome results revealed that the proportion of *C*Las colonization differed among samples from different sources, potentially due to host genetic differences driving *C*Las colonization. Genetic variations were identified through whole‐genome sequencing (WGS). The sequencing produced 460.48 million clean reads with an average mapping rate of 73.58% to the reference genome (Table S5). After SNP calling and filtering, 1,593,389 SNPs were obtained and used for subsequent analysis.

**FIGURE 3:**
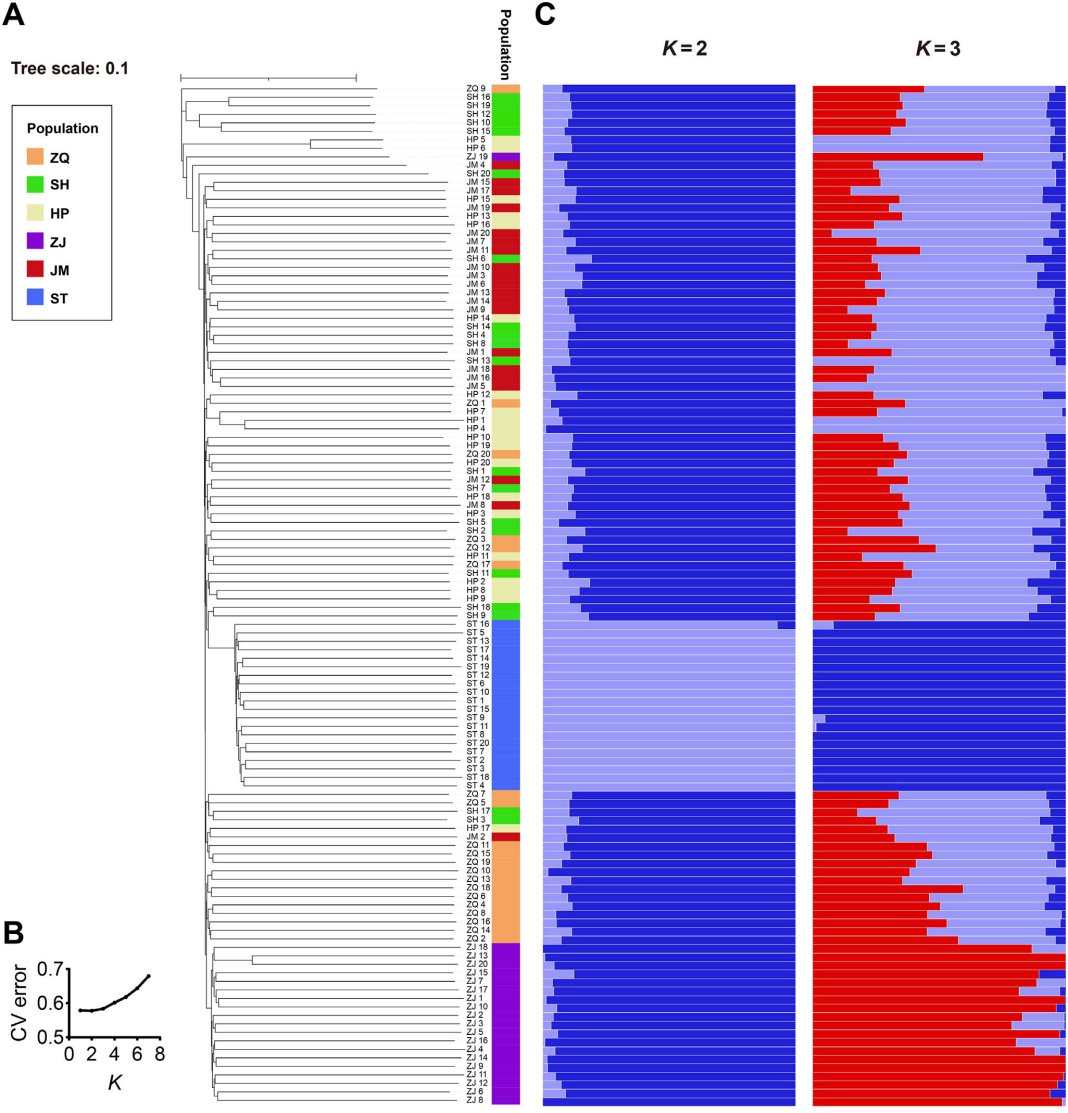
Phylogeny and population structure of *D.citri* populations. (A) Phylogenetic tree of *D.citri* population. Different color bars indicate different populations. (B) Cross‐validation (CV) error calculated using ADMIXTURE software. (C) Structure of *D.citri* populations inferred by ADMIXTURE software. Each color represents one subpopulation. Each individual is represented by a bar, and the length of each colored segment in the bar represents the proportion contributed by that subpopulation.

We estimated the phylogenetic relationships of all *D. citri* populations using the filtered SNPs. Phylogenetic analysis revealed distinct clades for the ST and ZJ locations (except for sample ZJ‐19) (Figure 3A), in eastern and western Guangdong provinces, respectively. Structural analysis also revealed genetic distinctions between the ST and ZJ populations and other populations (Figure 3B,C). When *K* = 2, two separate clusters (ST and the other populations) were observed, whereas when *K* = 3, three separate clusters (ST, ZJ, and the other populations) were observed.

**FIGURE 4:**
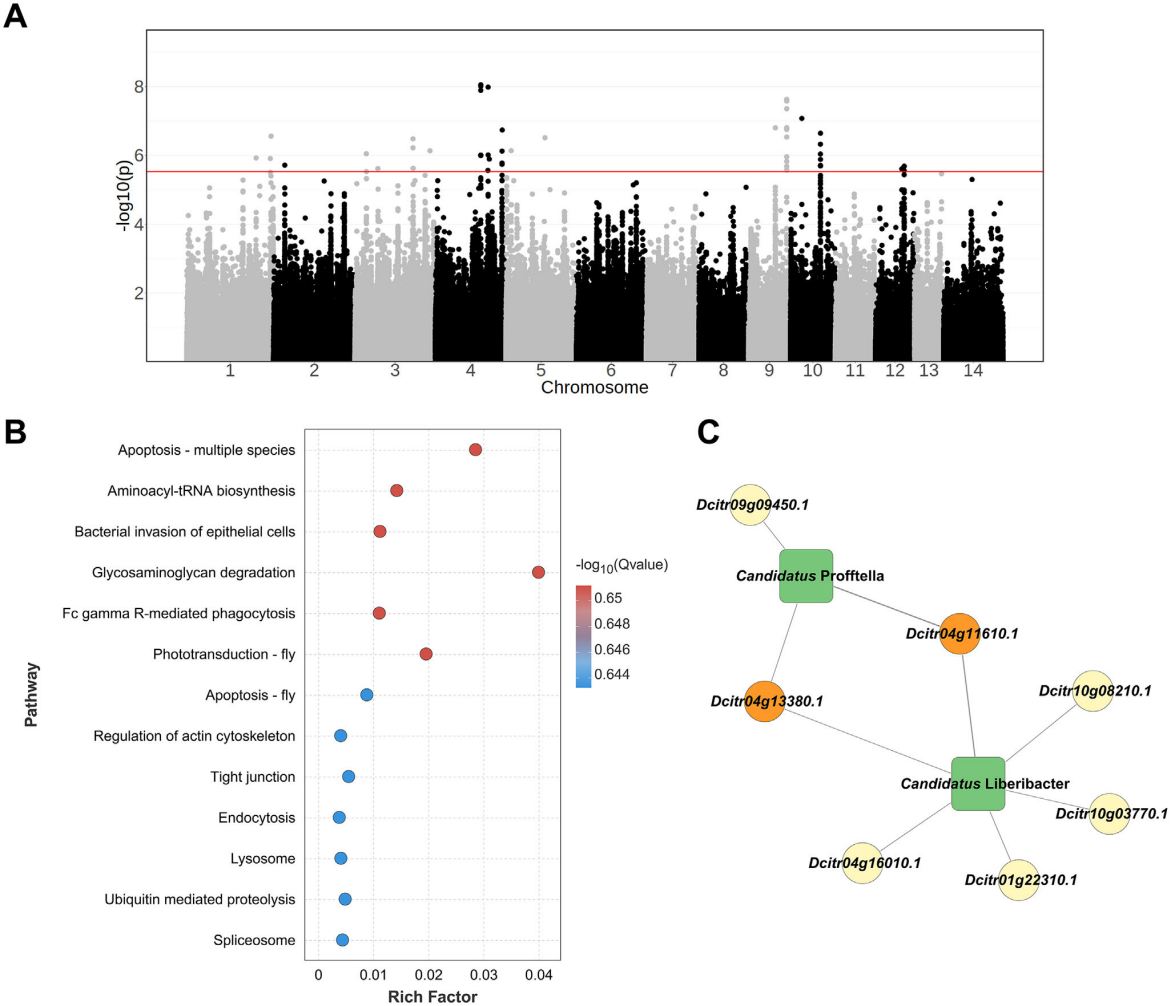
Genetic variations associated with bacterial diversity and *Candidatus* Liberibacter abundance. (A) A Manhattan plot of genome‐wide associations between genetic variations and the relative abundance of *C*Las. SNPs above the red line were considered significant (single nucleotide polymorphism) SNPs (Benjamini and Hochberg adjusted *p* value < 0.05). (B) KEGG enrichment of the genes containing significant SNPs associated with the relative abundance of *Candidatus* Liberibacter is analyzed. (C) Gene‐microbiota interaction network related to *Candidatus* Liberibacter in *D. citri*. The node shape of a square represents microbiota, and a circle represents a gene. The node colors of genes are varied based on the number of functional SNPs in the corresponding gene.

Thereafter, mGWAS was performed to determine the association between host insect genetics and the relative abundance of *C*Las in *D. citri* using genetic kinship as a random effect. Here, 79 genome‐wide SNPs belonging to 21 gene loci were identified to be significantly associated to the relative abundance of *C*Las (Figure 4A; Table S6). Notably, approximate half of the significant SNPs were clustered on the chromosome four and located at the regions of seven genes: *Dcitr04g11610.1* (MFS‐type transporter), *Dcitr04g11620.1* (unknown protein), *Dcitr04g11630.1* (neither inactivation nor afterpotential protein C), *Dcitr04g13380.1* (mediator of RNA polymerase II transcription), *Dcitr04g13400.1* (sugar transporter), *Dcitr04g16010.1* (unknown protein), and *Dcitr04g16090.1* (unknown protein). Further functional analyses revealed that candidate genes associated with the relative abundance of *C*Las were involved in various immunity‐related pathways, such as apoptosis, bacterial invasion of epithelial cells, endocytosis, and lysosomes (Figure 4B; Table S7).

To better understand the gene‐microbiota interaction network in this insect, GWAS was conducted to identify the gene loci associated with the relative abundance of core symbionts *Candidatus* Profftella and *Wolbachia* in *D. citri* (Figure S2 and Table S8). Forty‐seven SNPs were significantly associated with the relative abundance of *Candidatus* Profftella, and only one SNPs located in an intergenic region were significantly associated with the relative abundance of *Wolbachia*. To exclude the noise of nearby neutral hitchhiker loci, we retained only those gene loci affected by variants with potential functional changes (missense variants, upstream gene variants, 5’‐ and 3’‐ UTR variants, and splice region variants). An interaction network was then constructed to link the genes and microbiota based on their correlation (Figure 4C). The relative abundance of *C*Las was associated with six genes, among which *Dcitr04g11610.1* contained the largest number of functional SNPs. Notably, *Dcitr04g11610.1* was also associated with the relative abundance of *Candidatus* Profftella.

### 2.3 Gene *Dcitr04g11610.1* was Associated with *C*Las Infectivity of *D. citri*

Our analysis showed the potential involvement of gene *Dcitr04g11610.1* in modulating the abundance of *C*Las and another important symbiont, *Profftella*, in *D. citri*. The genomic structure of this gene drew from annotation files, showed a total of ten exons comprising five transcripts with the same coding sequence in the entire conserved domain (Figure S3). The presence of start and stop codons in exons was confirmed through Sanger sequencing analysis (Figure S3). Further analysis showed that the SNPs with significant allele frequency differences between *C*Las‐positive and ‐negative samples included eight completely linked variants (five 5’‐UTR variants and three 3’‐UTR variants, pairwise *D*’ = 1.00) in *Dcitr04g11610.1* (*p* < 0.05, Fisher's test; Table S9 and Figure S4), which may have affected the expression levels of this gene. The reliability of all SNPs was validated using PCR amplification and Sanger sequencing (Figure S5).

**FIGURE 5:**
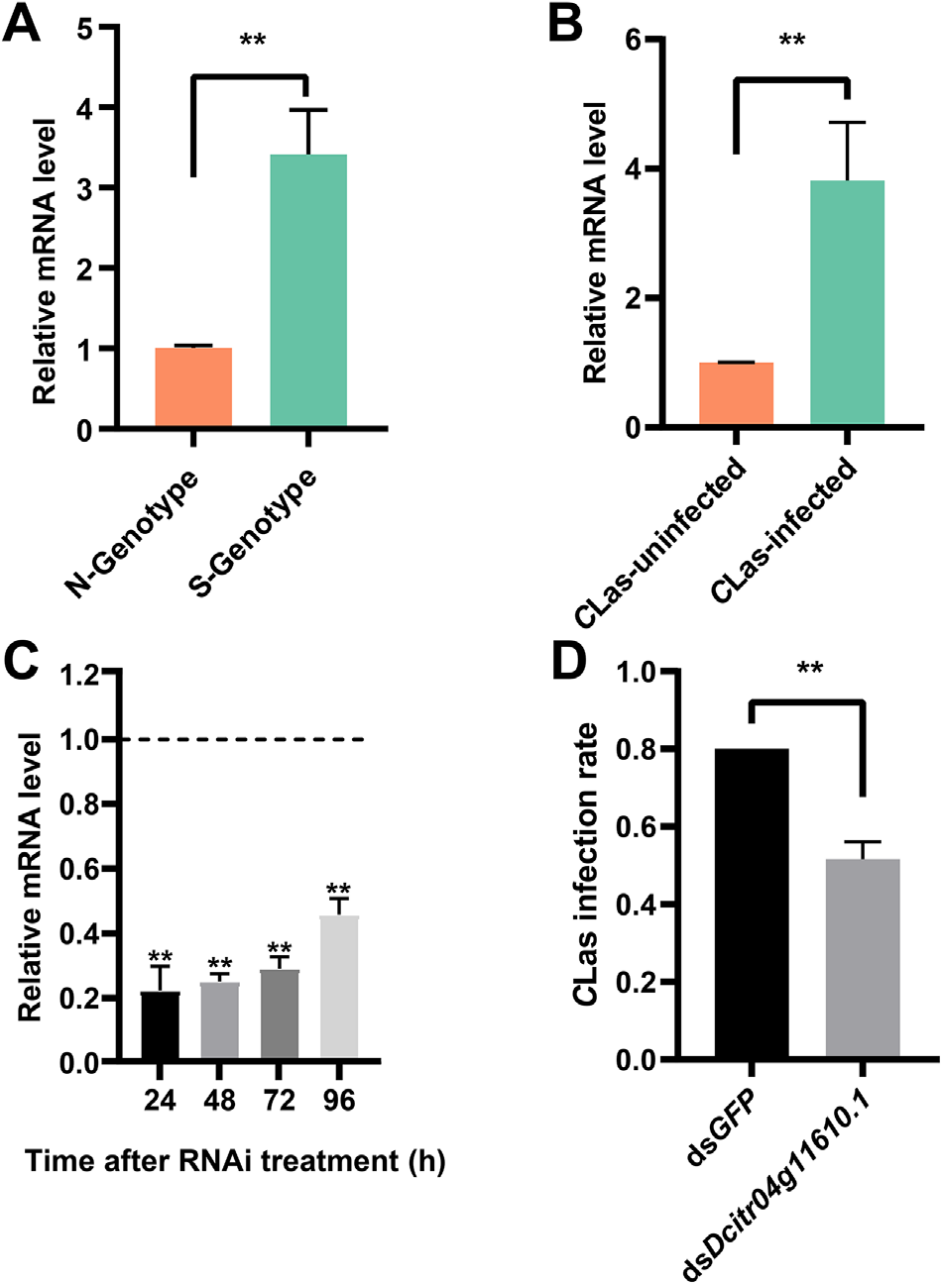
Functional validation of gene *Dcitr04g11610.1* in modulating *Candidatus* Liberibacter asiaticus (*C*Las) colonization in *D. citri*. (A) Genotype‐based expression of *Dcitr04g11610.1* in *C*Las‐uninfected samples. (B) Comparison of the expression of *Dcitr04g11610.1* in *C*Las‐infected and *C*Las‐uninfected samples of the laboratory population. (C) Relative expression of *Dcitr04g11610.1* in *D. citri* at 24, 48, 72, and 96 h after RNAi. The mRNA levels of insects treated with ds*GFP* were used as controls. (D) Change in *C*Las infection rate in *D. citri* fed with ds*Dcitr04g11610.1*. Data are shown as mean ± SEM (*n* = 4 for panel A, 3 for panel B and D, 18 for panel C), ^**^ indicates significant difference at *p* < 0.05 and *p* < 0.01 levels (Student's *t*‐test).

The association between genotype and the expression level of *Dcitr04g11610.1* was analyzed in *C*Las‐uninfected *D. citri* individuals of the ST population. Notably, individuals with the *C*Las‐susceptible genotype (S genotype) of *Dcitr04g11610.1* showed significantly higher mRNA levels than those with the normal genotype (N genotype) (*p* < 0.01, *t*‐test; Figure 5A). We further compared the expression levels of this gene between *C*Las‐infected *D. citri* and its *C*Las‐free counterparts in the laboratory population. The results showed that *Dcitr04g11610.1* was significantly more highly expressed in the *C*Las‐infected insects (Figure 5B).

The high expression of *Dcitr04g11610.1* may be beneficial for *C*Las infectivity in *D. citri*. To test this hypothesis, we knocked down the mRNA levels of *Dcitr04g11610.1* in *C*Las‐free *D. citri* using an RNA interference (RNAi) assay. The mRNA level of this gene was significantly reduced in insects fed with dsRNA 24 h post‐treatment compared to the ds*GFP* controls, and the reduction was most stable at 48 h (25.11%) but recovered to 45.83% at 96 h post RNAi treatment (Figure 5C). The fed insects were then transferred to citrus trees infected with *C*Las at 48 h post RNAi treatment. The infection rate of the transferred insects was tested after 36 h. Compared with insects fed with ds*GFP*, the *C*Las infection rate of insects fed ds*Dcitr04g11610.1* significantly reduced by 28 % (*p* < 0.01, *t*‐test; Figure 5D).

### 2.4 Gene *Dcitr04g11610.1* Impacted *C*Las Distribution and Titer in Infected *D. citri*

**FIGURE 6:**
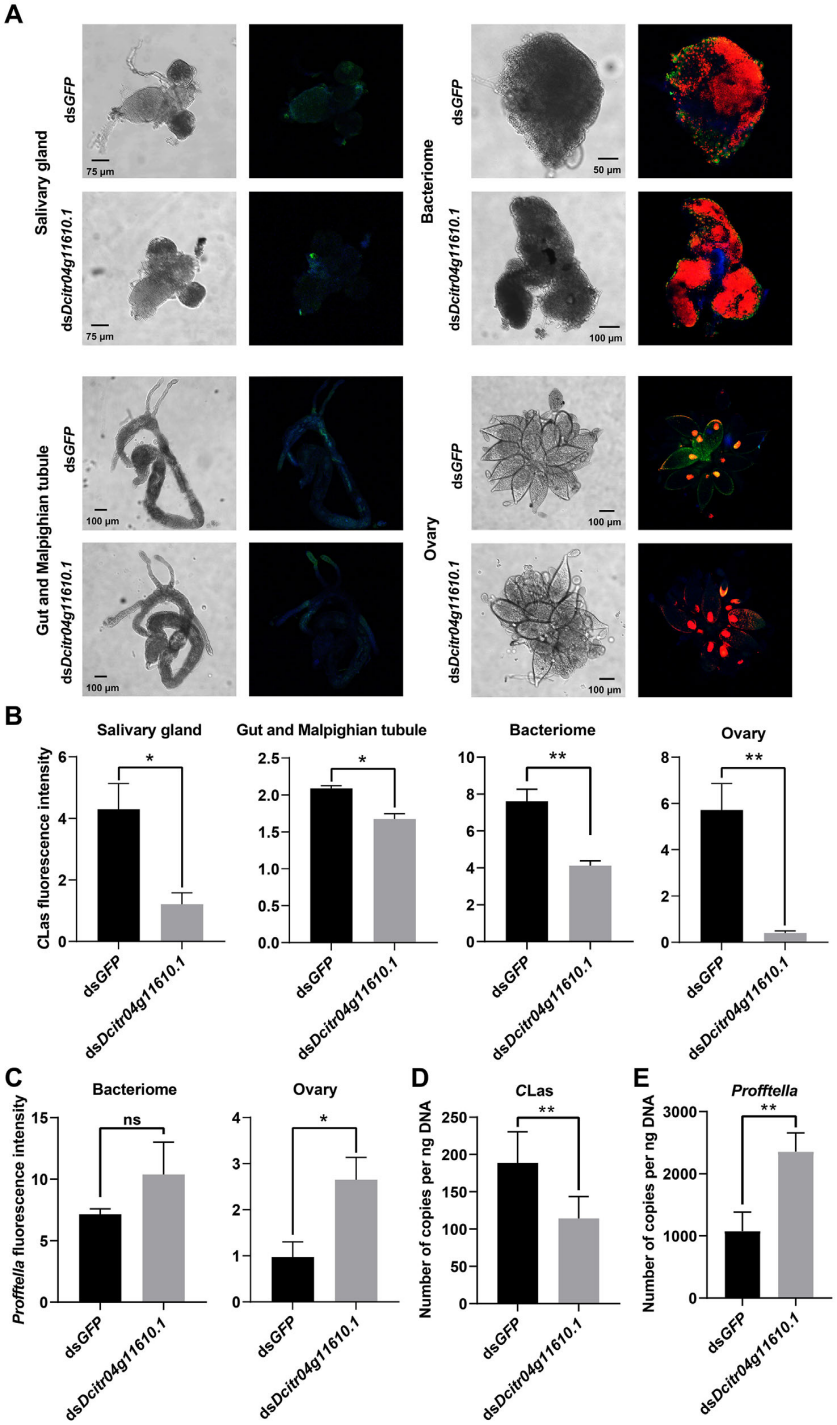
Gene *Dcitr04g11610.1* impacted *Candidatus* Liberibacter asiaticus (*C*Las) and *Candidatus* Profftella armatura distribution and titer in infected *D. citri*. (A) Representative confocal images of *C*Las and *Profftella* distribution in different tissues of *D. citri* samples fed with ds*GFP* and ds*Dcitr04g11610.1*. Cell nuclei are stained with DAPI and visualized in blue. *C*Las signal is visualized in green through staining with FAM. *Profftella* signal is visualized in red through staining with Cy3. (B) *C*Las fluorescence intensities in different tissues of *D. citri* samples fed with ds*GFP* and ds*Dcitr04g11610.1*. (C) *Profftella* fluorescence intensities in different tissues of *D. citri* samples fed with ds*GFP* and ds*Dcitr04g11610.1*. Copy numbers of *C*Las (D) and *Profftella* (E) in the infected samples fed with ds*Dcitr04g11610.1*, and ds*GFP* are detected. Data are shown as mean ± SEM (*n* = 3 for panel B and C, *n* = 9 for panel D and E), ^*^ and ^**^ indicate significant differences at *p* < 0.05 and *p* < 0.01 levels, respectively (Student's *t*‐test).

We further determined the impact of *Dcitr04g11610.1* knockdown on *C*Las abundance in infected *D. citri*. FISH was used to detect *C*Las signals in different tissues of *D. citri* samples, where *C*Las were reported to be mainly distributed in previous studies [26]. In insects fed ds*GFP*, *C*Las was abundant in the salivary gland, gut, Malpighian tubule, bacteriome, and ovary (green in Figure 6A). Whereas in insects fed ds*Dcitr04g11610.1*, the observed *C*Las signals were largely reduced, with fluorescence intensity significantly reduced by 71.76, 19.84, 45.88, and 92.98 % in salivary glands, guts with Malpighian tubules, bacteriomes, and ovaries, respectively (*p* < 0.05, *t*‐test; Figure 6B). Since *Dcitr04g11610.1* was also associated with the relative abundance of *Profftella*, the change in *Profftella* was also detected in bacteriomes and oocytes after RNAi targeting of this gene. Accordingly, the *Profftella* signal significantly increased in the oocytes (2.73‐fold in fluorescence intensity, *p* < 0.05, *t*‐test) but did not increase noticeably in the bacteriomes (1.45‐fold in fluorescence intensity, *p* > 0.05, *t*‐test) in insects fed ds*Dcitr04g11610.1* compared to those fed ds*GFP* (red in Figure 6A,C).

To further validate the changes in the abundance of *C*Las and *Profftella*, we used absolute quantitative PCR to detect the copy numbers of these two bacteria within *D. citri* samples. After ds*Dcitr04g11610.1* treatment, the copy number of *C*Las in the infected samples was significantly reduced by 39.56 %, compared with that in the ds*GFP* group (*p* < 0.01, *t*‐test; Figure 6D). By contrast, the knockdown of *Dcitr04g11610.1* significantly elevated the copy number of *Profftella* by 118.55 % in *D. citri*, compared with that in the ds*GFP* control (*p* < 0.01, *t*‐test; Figure 6E).

### 2.5 *Dcitr04g11610.1* Role in the Polyamine Utilization of *C*Las in *D. citri*

**FIGURE 7:**
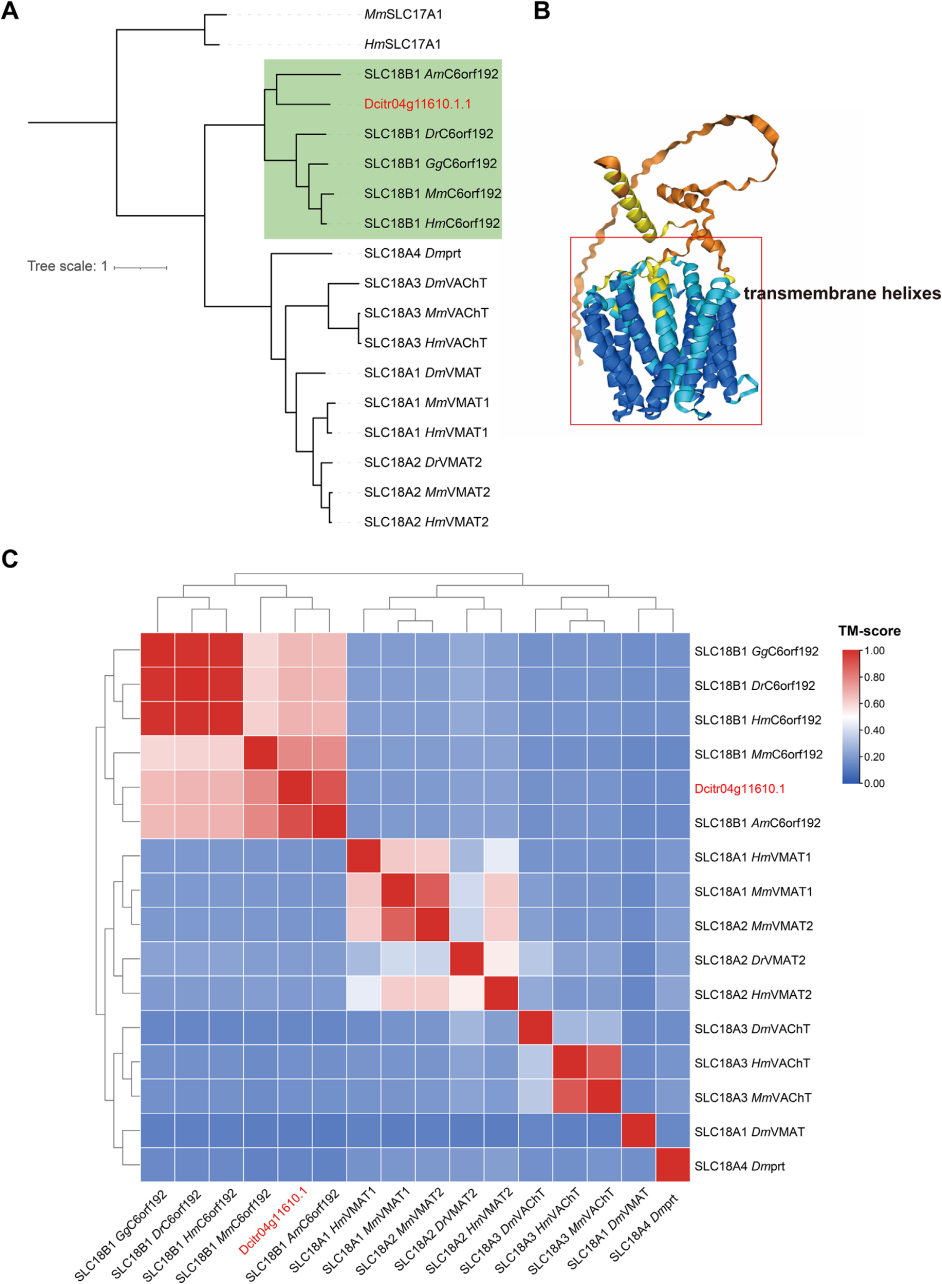
Analysis of gene *Dcitr04g11610.1* sequence and protein structure. (A) Maximum likelihood tree of *Dcitr04g11610.1* and SLC18 proteins in model animal species. The SLC17A1s from *H. sapiens* and *M. musculus* are used as outgroups. (B) The protein structure model of *Dcitr04g11610.1* was predicted using AlphaFold 3. The transmembrane domain is indicated. (C) Heatmap and the hierarchical clustering dendrogram illustrate the structural similarity among *Dcitr04g11610.1* and SLC18 proteins in model animal species.

The annotation from the NCBI Nr database showed that *Dcitr04g11610.1* was most homologous with the MFS‐type transporter SLC18B1‐like proteins found in the Hemiptera *Cyamophila willieti* and *Bemisia tabaci*. The phylogenetic analysis also revealed the evolutionary homology of *Dcitr04g11610.1* and other SLC18B1 in the model animal species (Figure 7A). The *Dcitr04g11610.1* protein structure predicted by AlphaFold 3 revealed no signal peptide but 12 transmembrane domains that conformed to the classical MFS protein structure (Figure 7B). The pairwise alignment and clustering of protein structures further confirmed the structural similarity of *Dcitr04g11610.1* and the SLC18B1 proteins in other species (Figure 7C). All these analyses validated that *Dcitr04g11610.1* belonged to the SLC18B1 subfamily rather than other SLC18 subfamilies.

**FIGURE 8:**
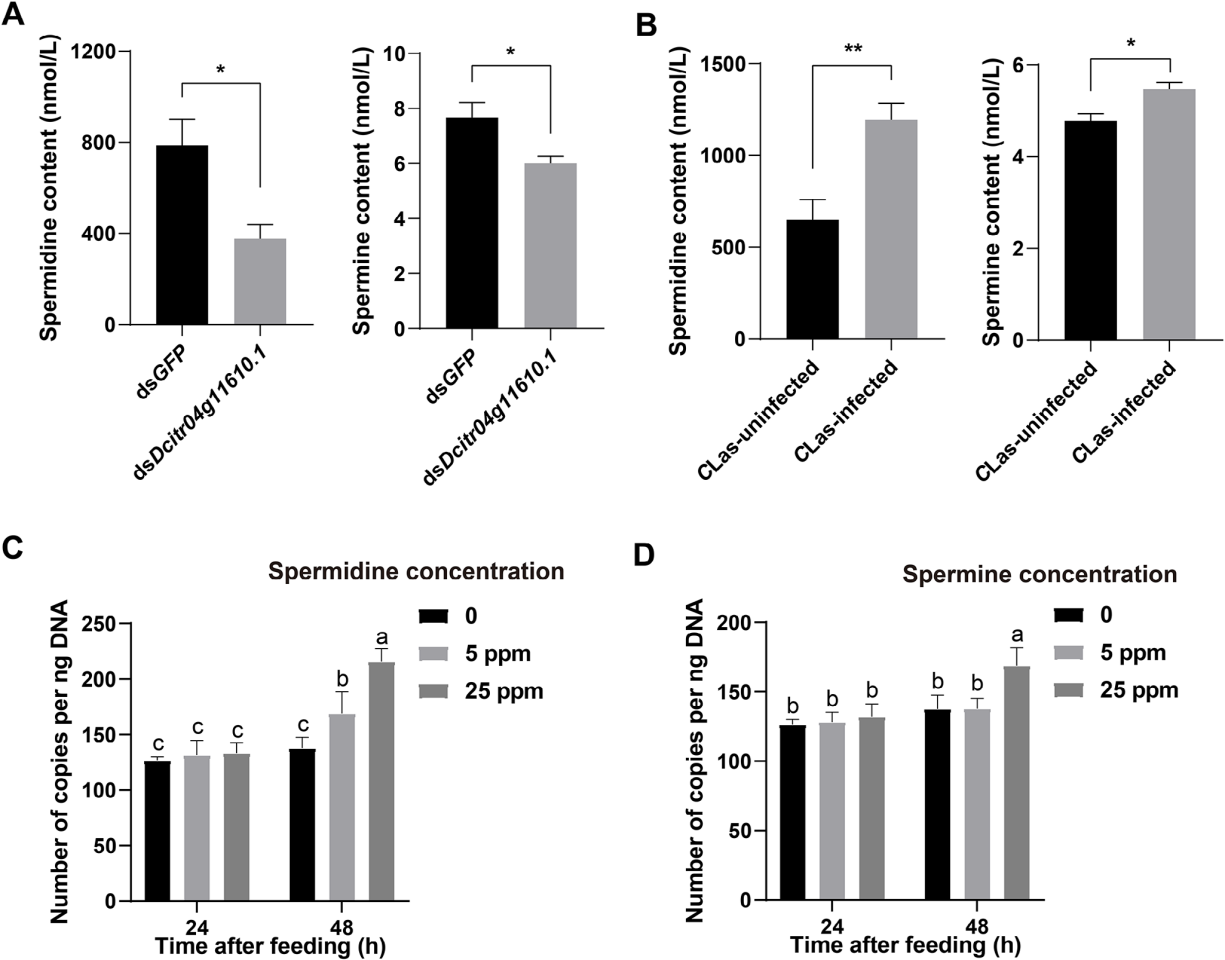
Validation on the relationship among *Dcitr04g11610.1*, polyamine transport, and *C*Las infection. (A) Extracellular spermidine and spermine abundances in the midgut of *D. citri* fed with ds*GFP* and ds*Dcitr04g11610.1*. (B) Extracellular spermidine and spermine abundances in midgut of *C*Las‐uninfected and ‐infected *D. citri*. The copy numbers of *C*Las in the infected samples fed with in artificial diets adding spermidine (C) and spermine (D) at 24 and 48 h. Data are shown as mean ± SEM (*n* = 4 for panels A and B, *n* = 3 for panels C and D). ^*^ and ^**^ in panels A and B indicate significant differences at *p* < 0.05 and *p* < 0.01 levels, respectively (Student's *t*‐test). The values sharing the same letter in panels C and D are not significantly different at *p* < 0.05 (Turkey's range test).

The function of SLC18B1 in mammals is mostly related to the exocytotic release of polyamines (spermidine and spermine) into the extracellular space [27, 28]. Here, we hypothesized that the function of SLC18B1 is conserved in animals. Based on this hypothesis, high expression of *Dcitr04g11610.1* meant that more polyamines were released extracellularly. Therefore, we detected the extracellular spermidine and spermine abundances in the midgut of *D. citri* fed with ds*Dcitr04g11610.1* through targeted metabolomics. Compared to the ds*GFP* control, the knockdown of *Dcitr04g11610.1* significantly reduced the extracellular spermidine and spermine contents by 52.05 and 21.69%, respectively (Figure 8A, *p* < 0.05, *t*‐test). Since the infection of *C*Las could lead to the overexpression of *Dcitr04g11610.1*, whether this process simultaneously leads to an increase in extracellular polyamine content was evaluated by targeted metabolomics. The results showed that the extracellular spermidine and spermine contents were significantly increased by 1.84 and 1.15‐fold after *C*Las infection (Figure 8B, *p* < 0.05, *t*‐test).

We further examined whether an increase in the amount of spermidine and spermine available in *D. citri* benefits *C*Las proliferation in the host insect, by adding spermidine and spermine into artificial diets, respectively. After feeding for 48 h, the addition of spermidine (5 and 25 ppm) and spermine (25 ppm) significantly elevated the copy number of *C*Las in infected *D. citri* compared to the control samples (Figure 8C,D).

## 3 Discussion

The acquisition of *C*Las by *D. citri* is mediated by the interaction of multiple factors related to the environment, vectors, and microbial symbionts. In this study, we focused on the intrinsic factors affecting the susceptibility of *D. citri* to *C*Las and explored the roles of symbiotic microbiota and genotype. Our findings provide evidence for the genetic basis of *C*Las acquisition in *D. citri*, as well as the potentially interacting effects of symbiotic microbiota and genotype.

Symbiotic bacteria in insects participate in host growth, development, and metabolism, as well as support host immunity and defense [29, 30]. The core symbiotic bacteria in *D. citri* include *Wolbachia*, *Profftella*, and *Candidatus* Carsonella ruddii [24, 31]. Our microbiome data showed that the bacterial taxonomy carried by different genetic backgrounds of *D. citri* in Guangdong was relatively conserved, with *Profftella* being the dominant genus, accounting for an average of 69.83% of the total samples, followed by *Wolbachia* with an average of 23.43% (Figure 2). *Carsonella* was also present in all the populations, but at a relatively low abundance (Table S4). This may be due to the mismatches in the primers (338F and 806R) used for the amplicon sequencing against the *Carsonella* genome, biasing the PCR amplification efficiency for the 16S rRNA gene of *Carsonella* [32, 33]. Owing to the limitation of primer bias, this study did not reflect well the potential interactions between *Carsonella* and *C*Las and with host insect genetics. However, these primers were efficient in amplifying endosymbiont *Profftella* and *C*Las in *D. citri* [32]. Especially for *C*Las, our sequencing detected the infection of *C*Las in all samples, although only approximate 20% of these samples were potentially colonized by *C*Las; the majority of uncolonized individuals could not be detected using conventional PCR. The mGWAS based on the relative abundance of *C*Las also successfully yielded desirable candidate genes.

*D. citri* has a defective innate immune system and lacks the IMD pathway to recognize Gram‐negative bacteria [34, 35] However, microbial symbionts may assist host defence by synthesizing a variety of bioactive substances to resist infection [36]. The symbiotic bacteria in *C*Las‐uninfected *D. citri* were mainly enriched in the nutrient synthesis pathway, such as amino acids and vitamins, whereas the symbionts in *C*Las‐infected *D. citri* were mainly related to the immune system pathway [37], suggesting immunological shifts in *D. citri* when infected with *C*Las. Previous studies have shown that the dominant symbiotic bacterium, *Profftella*, has potential interplay with *C*Las [38]. *Profftella* is a symbiotic defense bacterium that produces diaphorin with inhibitory effect on fungi [39]. Furthermore, *Profftella* can unite with the symbiotic bacterium *Carsonella* to form defense‐related bacteriocytes [26]. Therefore, the successful colonization of *C*Las in *D. citri* may depend, to some extent, on the change in the abundance of *Profftella*.

Genetic background is also considered an important factor that influences the susceptibility of the vector insect [3]. Field populations of *D. citri* have shown high levels of genetic diversity, as reported for different populations in southwestern and southern China [40]. Our results indicate the existence of at least two subpopulations within the tested populations (Figure 4). Among these populations, the ST population contained the highest number of *C*Las‐colonized individuals (10/20), followed by the ZJ population (9/20). Notably, both ST and ZJ showed significantly different genetic structures from each other and the other tested populations, according to the clustering results. This supports previous findings indicating that different *D. citri* lineages vary in their acquisition and transmission rates of *C*Las [13, 41].

We used an mGWAS to determine the genetic variations associated with the susceptibility of *D. citri* to *C*Las. mGWAS has been widely applied in identifying the genetic determinants of gut microbiota in humans, poultry, and livestock [42, 43, 44, 45]. Here, we identified 79 SNPs associated with the relative abundance of *C*Las. The genes related to these SNPs are involved in multiple immune‐related pathways, such as apoptosis, bacterial invasion of epithelial cells, endocytosis, and lysosomes. Apoptosis is one of the main defense mechanisms against pathogens and is responsible for eliminating damaged and pathogen‐infected cells and maintaining the function of the cellular immune system [46]. Apoptosis has also been observed in insect vectors infected with plant viruses [47]. Endocytosis and lysosomes are frequently associated with immune responses against pathogen infections [48, 49]. *C*Las manipulates host insect genes to obtain nutrients from *D. citri*. The *C*Las genome contains a functional adenosine triphosphate‐adenosine diphosphate (ATP/ADP) transcriptase that can be utilized by *C*Las to obtain ATP from *D. citri*. This is achieved by stimulating the ATP synthase subunit in *D. citri* to synthesize more ATP and a variety of other high‐energy nucleotides, while inhibiting ATP consumption by the insects [50, 51]. *C*Las also secretes effectors to counteract host insect defense and immune activities and colonize as quickly as possible [52]. The Sec secretion system of *C*Las produces Sec translocon (SEC)‐dependent effectors, most of which are expressed in *C*Las‐infected *D. citri* [52]. Therefore, how these effects interact with genes involved in immune‐related pathways in *D. citri* deserves in‐depth exploration in future studies.

We further found several significant SNPs located in the regulatory regions of gene *Dcitr04g11610.1* (5’‐UTR‐ and 3’‐UTR) and validated that these SNPs were associated with the overexpression of *Dcitr04g11610.1* in *C*Las‐susceptible individuals of *D. citri* (Figure 5A). Notably, RNAi knockdown of *Dcitr04g11610.1* significantly reduced the *C*Las‐infection rate and *C*Las abundance in infected *D. citri* (Figures 5D, 6). Phylogenetic and structural analyses suggest that *Dcitr04g11610.1* encodes an SLC18B1 protein, a recently identified member of the vesicular amine transporter family. SLC18B1 is broadly conserved across the animal kingdom and, in mammals, facilitates exocytotic release of polyamines (spermidine and spermine) into the extracellular space [27, 28]. Unlike other SLC transporters restricted to neurons and neuroendocrine cells, SLC18B1 is widely expressed across diverse cell types [27, 53, 54]. Although its role in insects remains uncharacterized, structural similarity implies functional conservation. Our target metabolomics results demonstrated that elevated expression of *Dcitr04g11610.1* may enhance extracellular polyamine release (Figure 8), and such a correlation might be exploited by *C*Las, which lacks polyamine biosynthetic pathways and may rely on its insect host for supply [55]. Supporting this, polyamine supplementation promoted *C*Las proliferation in infected *D. citri*, and transcriptomic data show significant up‐regulation of *Dcitr04g11610.1* in response to infection [56, 57]. Similar polyamine induction in *C*Las‐infected citrus plants further suggests that polyamines are critical for *C*Las survival, possibly explaining its inability to grow in vitro [58].

The gene‐microbiota interaction network in our study indicated that *Dcitr04g11610.1* was statistically associated with *Profftella*, and the knockdown of *Dcitr04g11610.1* resulted in a significant increase in the copy number of *Profftella* in *D. citri*, which was the inverse of the trend with *C*Las abundance (Figure 6). Previous studies have shown that *Profftella* has established a stabilizing symbiotic relationship with *D. citri* by forming the bacteriocytes [26, 59]. The symbiotic relationship is based on the metabolic complementarity between both organisms, especially since *Profftella* has a reduced genome that lack of most genes related to amino acid synthesis [60]. Therefore, *Profftella* may compete with *C*Las for nutrients in infected *D. citri*, as was implied in previous studies [38]. Based on this inference, the knockdown of *Dcitr04g11610.1* in infected *D. citri* reduced the proliferation of *C*Las and instead increased the resources available to *Profftella*. The elevated abundance of *Profftella* might in turn have negative effects on *C*Las proliferation in *D. citri*. Limited by the lack of CRISPR gene editing technology in *D. citri* at the current stage, the above hypothesis will be clarified and validated in more geographical populations in future investigations. In addition, the relationship between *Dcitr04g11610.1* and *Profftella* in this study represents a statistical association derived from GWAS. However, how *Dcitr04g11610.1* participates in the interaction between *C*Las and *Profftella* still needs to be further determined.

In summary, this study constructed a gene‐microbiota interaction network related to *C*Las susceptibility in the HLB vector *D. citri* using an mGWAS strategy. An important gene, *Dcitr04g11610.1* were identified, and functional validation showed that this gene may modulate *C*Las infection in *D.citri* and impact the abundance of *Profftella*, a key endosymbiont of *D. citri*, possibly by regulating the polyamine transport. The identified SNPs associated with *C*Las infectivity can be used as molecular markers for future detection of *C*Las‐susceptible populations. Furthermore, our findings broaden our understanding of the role of microbial and genetic factors in pathogen and vector insect interactions, which will facilitate efficient prevention and management of *C*Las in the future.

## 4 Experimental Section

### 4.1 Insects

To minimize the effect of biases in time (climate change in different seasons or across years) on the detection of *C*Las infection rate [61], we sampled the field *D. citri* between March and May 2021. *D. citri* female adults from field populations were collected using an aspiration (“poota”) device from citrus orchards in six different regions of Guangdong, China (Figure 1). The six geographic populations were reared on *Citrus sinensis* L. Osbeck (HP population), *Citrus reticulata* cv. Chachiensis (JM population), *C. reticulata* Blanco cv. Shiyue Ju (SH population), *C. reticulata* var. tankan Hayata (ST population), *C. sinensis* Osbeck cv. Hongjiang (ZJ population), and *C. reticulata* Blanco var. Gonggan (ZQ population). Each population included more than 100 individuals collected from *C*Las‐infected citrus trees in at least three orchards in the region. After sampling, insects were individually and immediately immersed and stored in anhydrous ethanol for subsequent genomic DNA extraction.

Additional insects from ST population sampling from healthy citrus trees in orchards that did not show any symptoms of *C*Las infection were used for testing the association between genotypes and gene expression. A laboratory *D. citri* population used for functional validation was kept in our lab from 2019 and had been continuously reared for more than 20 generations in an artificial climate chamber with a mixture of healthy *C. reticulata* Blanco cv. Shiyue Ju and *Murraya exotica* L., and this population was tested and confirmed to be *C*Las‐free. The temperature, relative humidity (RH), and photoperiod were 27 ± 1°C, 70 %, 14 L: 10 D, respectively.

### 4.2 DNA Extraction, PCR Amplification, and Illumina Sequencing

Total DNA was extracted from the whole body of approximately 100 individuals from each population using a DNA extraction kit (HiPure Blood DNA Mini Kit; Magen Biotechnology Co., Ltd., Guangzhou, China), following the manufacturer's instructions. All samples underwent the following cleaning procedure before DNA extraction to eliminate potential surface contamination: surface‐sterilized with 70% ethanol for 1 min, 10% sodium hypochlorite for 1 min, and three washes of ultrapure water for 1 min. Quality control of the extracted DNA was conducted using a Qubit 4.0 Fluorometer (Thermo Fisher Scientific Inc., Waltham, MA, USA). The resulting DNA samples were used for the detection of *C*Las infection using the conventional PCR primers OI1 and OI2c specific to a 1160 bp fragment of 16S rDNA for *C*Las as previously described [15, 62]. Based on the infection rates obtained by the conventional PCR detection (Figure 1), we randomly selected 20 individuals, including both PCR‐positive and ‐negative samples in each population, for subsequent high‐throughput sequencing (Table S1).

Part of the remaining DNA of selected samples was used to amplify the V3–V4 hypervariable regions of the prokaryotic 16S rDNA with the primers 338F (5’‐ACTCCTACGGGAGGCAGCA‐3’) and 806R (5’‐GGACTACHVGGGTWTCTAAT‐3’) as described in previous studies [63, 64]. PCR amplification was performed in triplicate; each reaction volume contained 2.5 µL of TransStart Buffer (TransGen, Beijing, China), 2.5 µL of TransStart Taq DNA polymerase (including 2 µL of dNTPs), 1 µL of each primer (10 µm), and 20–30 ng of template DNA in a total reaction volume of 25 µL. Index adapters were connected to the ends of the amplicons to generate the indexed libraries with ALFA‐SEQ DNA Library Prep Kit (Finorop, Guangzhou, China) according to the manufacturer's instructions. Quality control of the PCR products with index adapters was performed using Qseq400 (BiOptic Inc., New Taipei, Taiwan), and 10 nmol of the product was used for subsequent next‐generation sequencing. The DNA libraries were loaded onto the Illumina MiSeq platform (San Diego, CA, USA), and subsequent sequencing was performed using the paired‐end 250‐bp mode at MAGIGENE (Guangzhou, China). Cutadapt software was used to trim raw reads to generate clean reads.

### 4.3 Taxonomic Classification and Microbial Diversity Analysis

Clean reads were processed using the QIIME2 pipeline [65]. The “demux” plugin was used to inspect the paired‐end sequencing reads. The reads were then processed using the DADA2 pipeline to trim reads, correct errors, merge read pairs, and remove PCR chimeras to obtain amplicon sequence variants (ASVs) and representative sequences with quality settings “–p‐trunc‐len‐f 228” and “–p‐trunc‐len‐r 214” [66]. The obtained ASVs were taxonomically classified using the Greengenes database release 13_5 and the Silva database release 138. ASVs assigned to chloroplasts, mitochondria, and singletons across all samples were filtered, and only ASVs assigned to bacteria were retained for subsequent analysis.

### 4.4 Genome Resequencing and Variant Calling

The remaining DNA was used for amplicon sequencing and genome resequencing. After quality checking, the DNA samples were randomly fragmented into 300‐bp fragments and used for DNA library construction. DNBs (DNA Nano Bail) were prepared after passing the library inspection and then loaded onto the sequencing chip for paired‐end (PE)‐150 bp sequencing using the MGI High‐Throughput Sequencer platform (BGI, Shenzhen, China) at Frasergen (Wuhan, China).

Quality control of raw reads was conducted using SOAPnuke with parameters “–lowQual = 20, –nRate = 0.005, –qualRate = 0.5” (ver 2.1.0). Clean reads were mapped to the reference genome of *D. citri* (V3.0, https://citrusgreening.org/organism/Diaphorina_citri/genome) using BWA mem (ver 0.7.15) [67]. The Genome Analysis Toolkit (GATK, ver 4 2.0) was used to sort the BAM files and remove reduplicates [68]. SNP calling and genotyping were performed using the Haplotype Caller module in GATK. To ensure accuracy in variant calling, single nucleotide polymorphism (SNPs) within 5 bp of an INDEL (insertion or deletion) and SNP clusters (more than three SNPs within a 10 bp window) were removed. The variant call format (VCF) files of SNPs were further combined and filtered with the VariantFiltration module in GATK using the threshold of “QD < 2.0 || MQ < 40.0 || FS > 60.0 || SOR > 3.0 || QUAL < 30”. Because alleles at lower frequencies are less informative for association analysis, software VCFTools (version 0.0.16) was used to filter SNPs with minor allele frequencies (MAFs) below 5 % and Hardy–Weinberg Equilibrium's *p*‐value < 0.01. We retained only SNPs that occurred in more than 90 % of individuals. The final SNPs were further annotated using the snpEff software ver 4.3 [69].

### 4.5 Phylogenetic Analysis and Population Structure Inference

The filtered VCF file containing the final SNPs was transferred to a distance matrix using the VCF2Dis software (https://codeload.github.com/hewm2008/VCF2Dis). The distance‐based phylogeny was then inferred from the matrix file using FastME 2.0 [70]. iTOL (https://itol.embl.de) was used to annotate the phylogenetic trees.

SNPs were pruned using PLINK (version 1.90) with a window size of 50, step size of 10, and r^2^ threshold of 0.5. Population structure was analyzed using ADMIXTURE (version 1.3.0) [71, 72]. To obtain the convergence of individuals, we predefined the number of genetic clusters (*K* value) from 1 to 7 and ran a cross‐validation error (CV) procedure with default parameters.

### 4.6 GWAS

GWAS was performed to detect SNPs affecting bacterial diversity and abundance of microbial genera using the linear mixed model in GEMMA (version 0.98.3) [73]. The beta diversity and log_10_‐transformed abundance of specific microorganisms were used as input phenotypes. Genetic kinship inferred from the filtered VCF file was used as a random effect. The *p‐values* of the SNP effects were calculated using the likelihood ratio test and adjusted according to the Benjamini and Hochberg method. The genome‐wide significance threshold was set at an adjusted *p‐value* of < 0.05.

To genotype candidate SNPs, PCR amplification was conducted using the KAPA2G Fast Genotyping mix according to the user guide (KAPA, Boston, MA, USA) with specific primers targeting the corresponding genomic regions (Table S10). The PCR products were sent to Sangon Biotechnology Ltd. (Guangzhou, China) for Sanger sequencing. The sequencing chromatograph was analyzed using the Chromas software (www.technelysium.com.au). The linkage disequilibrium of the candidate SNPs was analyzed using Haploview (version 4.2) [74].

### 4.7 Construction of Gene‐Microbiota Interaction Network

The significant SNPs were attributed to the gene loci according to the snpEff annotation, and only those genes containing variants with potential functional changes (missense variants, upstream gene variants, 5’‐ and 3’‐untranslated region (UTR) variants, and splice region variants) were retained for further analysis. Next, the gene‐microbiota relationship network was constructed based on the genes containing significant SNPs associated with the abundance of specific microbiota. These gene‐microbiota relationships were linked to the microbial co‐occurrence network and visualized using Cytoscape software.

### 4.8 Comparison of *Dcitr04g11610.1* Expression Across Genotypes and Infection Conditions

To determine the association between genotypes and expression of gene *Dcitr04g11610.1*, the ST population collected from healthy citrus trees was reared on healthy citrus trees in the laboratory for one generation. After determining that the offsprings were all *C*Las‐uninfected using conventional PCR as described above and the citrus trees did not show the symptoms of *C*Las infection, total DNA and RNA were co‐extracted from the offsprings of this population using an Ultra‐Micro Tissue Cell RNA/DNA extraction Kit (MIKX, Shenzhen, China). The extracted DNA served as a template for PCR amplification, with specific primers targeting significant SNPs in gene *Dcitr04g11610.1* (Table S10). The resulting PCR products were then genotyped using Sanger sequencing. RNA was used for cDNA synthesis through the transcript. One gDNA Removal and cDNA Synthesis Super Mix (TranGen Biotech, Beijing, China) according to the manufacturer's instructions. The primers used for real‐time quantitative PCR (qPCR) detection of *Dcitr04g11610.1* are listed in Table S10. qPCR was performed on Quantitative PCR Instrument qTOWER3 (Analytik Jena AG, Germany) using PerfectStart Green qPCR SuperMix (TranGen Biotech, Beijing, China) with the following conditions: 94°C for 30 s followed by 40 cycles of 95°C for 5 s, 60°C for 15 s, and 72°C for 10 s. The *β‐Actin* gene was used as an endogenous control, and the gene expression level was calculated using the 2^–△△^CT method.

*C*Las‐free laboratory population samples were transferred to *C*Las‐infected citrus plants and continuously reared for six generations, and the expression levels of candidate genes in the *C*Las‐infected progenies were compared with those in *C*Las‐free individuals. Total DNA and RNA were extracted from the same individuals, as earlier described. DNA was used to detect *C*Las infection by conventional PCR, and the results showed that all tested samples reared on *C*Las‐infected plants were *C*Las‐positive. RNA was used for subsequent qPCR analysis of gene expression.

### 4.9 RNA Interference and Infection Assay

The template for the double‐stranded RNA (dsRNA) synthesis was amplified using primers containing the T7 promoter sequence at both ends (Table S10). The dsRNA of gene *Dcitr04g11610.1* was synthesized using the RiboMax Express RNAi System (Promega, Madison, WI, USA), following the manufacturer's instructions. The dsRNA was then added to the artificial diet to achieve a final dsRNA concentration of 150 ng/µL. Using the same method, ds*GFP* was synthesized in parallel and added to an artificial diet at an equal concentration to the control.

We used the feeding method for RNA interference (RNAi) in *D. citri*. Newly emerged female adults from the *C*Las‐free laboratory population were collected and starved for 6 h. Subsequently, the insects were transferred to glass cylinders sealed with stretched Parafilm M (Pechiney Plastic Packaging Company, Chicago, IL, USA) at both ends. Artificial feed containing dsRNA was added between two layers of stretched Parafilm M. Treatment for each of the 30 individuals was conducted in triplicate. The interference efficiency of the target genes was determined with qPCR after 48 h of feeding. Insects fed with ds*GFP* were used as controls. An infection assay was conducted on the surviving insects after feeding them with dsRNA for 48 h. The insects were then transferred to *C*Las‐infected citrus trees and fed continuously for 36 h. The *C*Las infection rate in the treated samples was determined using PCR amplification.

### 4.10 Determining the Effects of RNAi Knockdown of *Dcitr04g11610.1* on *C*Las Distribution and Titer in Infected *D. citri*

*C*Las‐infected *D. citri* from the laboratory population described above continued to be placed on *C*Las‐infected citrus trees for feeding over one generation. Female adults from the next‐generation were selected and fed with dsRNA, as earlier described. After 48 h, salivary glands, guts with Malpighian tubules, bacteriomes, and ovaries dissected from *D. citri* samples treated with dsRNA were collected for fluorescence in situ hybridization (FISH) to observe the distribution of *C*Las and *Profftella* using the method described by Hosseinzadeh [75]. The tissue samples were firstly fixed using Carnoy^,^s fixative solution, which consists of chloroform, ethanol, and glacial acetic acid in a 6:3:1 volume ratio, with fixation performed for 5 min on sterile glass slides. The pre‐hybridization solution was added dropwise and incubated at 37 °C for 2 h. The pre‐hybridization solution was then removed, and a probe‐containing hybridization solution (5’‐FAM‐CATTATCTTCTCCGGCG‐3’ for *C*Las 16s rRNA, and 5’‐Cy3‐GACCCTCTGTATGCACCATT‐3’ for *Profftella* 16s rRNA) was added, followed by overnight incubation at room‐temperature (23°C). To remove the hybridization solution, the tissue samples were washed off with 2 × SSC at 40°C for 10 min, 1 × SSC at 40°C for 10 min, and finally with 0.5 × SSC at room‐temperature for 10 min. Next, 4',6‐diamidino‐2‐phenylindole (DAPI) staining solution was added to the tissue samples, followed by incubation in the dark for 8 min, and an anti‐fluorescence quenching sealing agent was added after washing. The prepared tissue samples were observed under a laser scanning confocal microscope (Leica Stellaris 5, Leica Microsystems, Germany), and images were collected at ultraviolet (UV) excitation wavelength of 330–380 nm and an emission wavelength of 420 nm (blue light). The green light excitation wavelength of the FAM (488) was 465‐495 nm, and the emission wavelength was 515–555 nm. For Cy3, the red light excitation wavelength was 510–560 nm, and the emission wavelength was 590 nm. The scanning settings were kept fixed across all experiments, and images were processed using Leica LAS‐AF software v4.7.0.

Confocal multichannel FISH images were quantified in ImageJ [75]. Briefly, channels were separated into blue (DAPI), green (*C*Las), and red (*Profftella*) single‐channel images using Color‐Split Channels. Regions of interest (ROIs) corresponding to specific fluorescence signals were defined in the target channel using the rectangular selection tool. For each ROI, Analyze‐Measure was used to obtain the ROI area and mean gray value. For channel‐wise normalization and between‐group comparisons, the mean gray value was measured for the same ROI in the red, green, and DAPI channels. Because an identical ROI (same position and area) was applied across all three channels, red and green fluorescence signals were normalized to the DAPI mean gray value within the same ROI to minimize variation arising from differences in nuclear density and minor imaging fluctuations. For each sample, normalized values from three ROIs were averaged to generate a representative value. Three replicate samples were measured for each group.

The insects fed with dsRNA were also collected for absolute quantitative PCR detection of the copy numbers of *C*Las and *Profftella* using the methods described by Dossi and Hosseinzadeh [76, 77]. The primers used for absolute quantitative PCR are listed in Table S10. The amplification efficiency of the primers and the standard curves and corresponding equations of absolute quantitative PCR are provided in Figure S6.

### 4.11 Phylogenetic and Structural Analyses of Gene *Dcitr04g11610.1*

The annotation of gene *Dcitr04g11610.1* was obtained by BLASTP in the NCBI Non‐redundant (Nr) database. The phylogenetic analysis was conducted for the amino acid sequences of gene *Dcitr04g11610.1* and the solute carrier 18 (SLC18) genes from model animal species *Homo sapien*s, *Mus musculus*, *Gallus gallu*s, *Danio rerio*, *Drosophila melanogaster*, and *Apis mellifera* using the SLC17A1s from *H. sapiens* and *M. musculus* as outgroup. The analyzed SLC18 genes included SLC18A1 (encoding vesicular monoamine transporter 1, VMAT1), SLC18A2 (encoding VMAT2), SLC18A3 (encoding vesicular acetylcholine transporter, VAChT), SLC18A4 (encoding portabella, prt), and SLC18B1 (encoding C6orf192). The amino acid sequences were aligned using mafft (version 7.52) [78], then the maximum likelihood (ML) tree was constructed using iqtree (version 2.2.2.9) with the best protein model of “Q.pfam+R3” and bootstrap 1000 times [79]. The signal peptide of *Dcitr04g11610.1* protein was predicted in SignalP 5.0 (https://services.healthtech.dtu.dk/services/SignalP‐5.0/). The transmembrane helices in *Dcitr04g11610.1* protein was predicted in TMHMM‐2.0 (https://services.healthtech.dtu.dk/services/TMHMM‐2.0/). The artificial intelligence (AI) software AlphaFold 3 (https://www.alphafoldserver.com) was used to generate 3D structural models of *Dcitr04g11610.1* and SLC18 family proteins of other model animals [80]. The online tool TM‐score (https://seq2fun.dcmb.med.umich.edu//TM‐score/) was used to calculate the pairwise similarity (TM‐score) of the transmembrane domain structure among these proteins [81]. The clustering heatmap of SLC18 protein similarity was drawn based on the TM‐scores.

### 4.12 Target Metabolomics Analysis of Extracellular Spermidine and Spermine Abundances in *D. citri*

For the detection of extracellular spermidine and spermine abundances, midgut tissues were dissected from *D. citri* adult female samples treated with dsRNA and samples infected with *C*Las. The dissected midgut was placed in 50 mm ammonium formate (pH 7.0) at a volume of 5–10 µL/mg per tissue, and was gently shaken at 4°C for 2–3 min. Then the mixture was centrifuged at 3000 × g, 4°C for 5 min, and the supernatant was collected. An equal volume of 80% methanol solution plus 0.1% formic acid was added to the supernatant. After vortexing and centrifuging at 12 000 × g for 10 min, the supernatant was collected. Derivatization was performed according to the previous report with a few modifications [82]. Briefly, a 100 µL aliquot of the supernatant was transferred to an Eppendorf tube, then mixed with 50 µL of 20 mg/mL dansyl chloride in acetonitrile, and 50 µL of 0.1 mol/L sodium bicarbonate, after 1 h incubation at 40°C in the dark. Dansyl derivatives were added into 50 µL of 1% formic acid in water, the samples were vortexed for 30 s, and centrifuged at 12 000 rpm (RCF = 13 800 × g, R = 8.6 cm) and 4°C for 15 min. A 100 µL aliquot of the clear supernatant was transferred to an auto‐sampler vial for UHPLC‐MS/MS analysis.

The UHPLC separation was carried out using an Agilent 1290 Infinity II series UHPLC System (Agilent Technologies), equipped with a Waters ACQUITY UPLC HSS T3 column (100 × 2.1 mm, 1.8 µm). The mobile phase A was 10 mmol/L ammonium formate and 0.1% formic acid in water, and the mobile phase B was acetonitrile. The column temperature was set at 35°C. The auto‐sampler temperature was set at 4°C and the injection volume was 2 µL.

An Agilent 6460 triple quadrupole mass spectrometer (Agilent Technologies), equipped with an AJS electrospray ionization (AJS‐ESI) interface, was applied for assay development. Typical ion source parameters were: capillary voltage = +4000/‐3500 V, Nozzle Voltage = +500/‐500 V, gas (N_2_) temperature = 300°C, gas (N_2_) flow = 5 L/min, sheath gas (N_2_) temperature = 250°C, sheath gas flow = 11 L/min, nebulizer = 45 psi. The MRM parameters for each of the targeted analytes were optimized using flow injection analysis, by injecting the standard solutions of the individual analytes into the API source of the mass spectrometer. Agilent MassHunter Work Station Software (B.08.00, Agilent Technologies) was employed for MRM data acquisition and processing.

Calibration solutions were subjected to UPLC‐MRM‐MS/MS analysis using the methods described above. The least squares method was used for the regression fitting. 1/x weighting was applied in the curve fitting since it provided the highest accuracy and correlation coefficient (R^2^). The level was excluded from the calibration if the accuracy of calibration was not within 80%–120%. The calibration standard solution was diluted stepwise, with a dilution factor of 2. These standard solutions were subjected to UHPLC‐MRM‐MS analysis. The signal‐to‐noise ratios (S/N) were used to determine the lower limits of detection (LLODs) and lower limits of quantitation (LLOQs). The LLODs and LLOQs were defined as the analyte concentrations that led to peaks with signal‐to‐noise ratios (S/N) of 3 and 10, respectively, according to the US FDA guideline for bioanalytical method validation. The precision of the quantitation was measured as the relative standard deviation (RSD), determined by injecting analytical replicates of a QC sample. The accuracy of quantitation was measured as the analytical recovery of the QC sample determined. The percent recovery was calculated as [(mean observed concentration) / (spiked concentration)] × 100%.

### 4.13 Determining the Effects Spermidine and Spermine on *C*Las Titer in Infected *D. citri*

The standard substances of spermidine (CAS:124‐20‐9) and spermine (CAS: 71‐44‐3) were purchased from Sigma. Spermidine and spermine were diluted into 0, 5, and 25 ppm (parts per million) in artificial diet. The feeding method was applied as above. After feeding for 24 and 48 h, the insects were collected and absolute quantitative PCR detection of the copy numbers of *C*Las.

### 4.14 Statistical Analysis

All statistical analyses were conducted using IBM SPSS Statistics 25 software (IBM, Armonk, NY, USA). All statistical data were shown as mean ± SEM (*n* ≥ 3). Differences in biological parameters between the two groups were analyzed using the Student's *t*‐test. Differences among multiple groups were compared using one‐way ANOVA, followed by Tukey's HSD test. Correlation between the presence of *C*Las in PCR detection and the relative abundance of *C*Las was calculated according to Pearson's correlation coefficient. Differences in allele frequency between groups were analyzed using Fisher's exact test.

## Author Contributions

K.L. and QC.H. contributed equally to this work. K.L., Q.C.H., Q.X.H., and R.P. designed research; K.L., Q.C.H., Z.L., M.G., and L.Z. collected the samples; K.L., Q.C.H., H.J., and G.W. performed the experiments; R.P., S.H., and Z.Z. performed bioinformatics analyses; K.L., P.Z., G.M.G., Q.X.H., and R.P. interpreted the data; K.L., G.M.G., Q.X.H., and R.P. wrote the paper; K.L. and Q.X.H. provide the fund.

## Conflicts of Interest

The authors declare no conflict of interest.

## Supporting information




**Supporting File 1**: advs74787‐sup‐0001‐SuppMat.docx.

**Supporting File 2**: advs74787‐sup‐0002‐Supplementary_tables.xlsx.

## Data Availability

The data that support the findings of this study are openly available in [NCBI SRA] at [https://dataview.ncbi.nlm.nih.gov/object/PRJNA1188454?reviewer=59mo4470821i248fq04rtjgual], reference number [240].
