# Supplementary material for: Genome‐Wide Association Study Reveals Insect Genetics and Microbial Symbiont Effects on Susceptibility of Diaphorina citri to the Citrus Greening Pathogen, Candidatus Liberibacter Asiaticus
Source: Adv Sci (Weinh). 2026 Mar 10;13(29):e17056. doi: 10.1002/advs.202517056 (PMC13205867; doi:10.1002/advs.202517056)
Supplement: Supplementary file 1 — Supporting File 1: advs74787‐sup‐0001‐SuppMat.docx. [file ADVS-13-e17056-s002.docx]

**Supporting Information**

**Genome-Wide Association Study Reveals Insect Genetics and Microbial Symbiont Effects on Susceptibility of *Diaphorina citri* to the Citrus Greening Pathogen, *Candidatus* Liberibacter Asiaticus**

*Kai Liu, Qingcui He, Zeyue Lin, Shixuan Huang, Zichun Zhong, Pingyang Zhu, Mengge Gao, Luyao Zhao, Han Jin, Guiting Wu, Gurr M. Geoff, Qunxin Han*, Rui Pang**


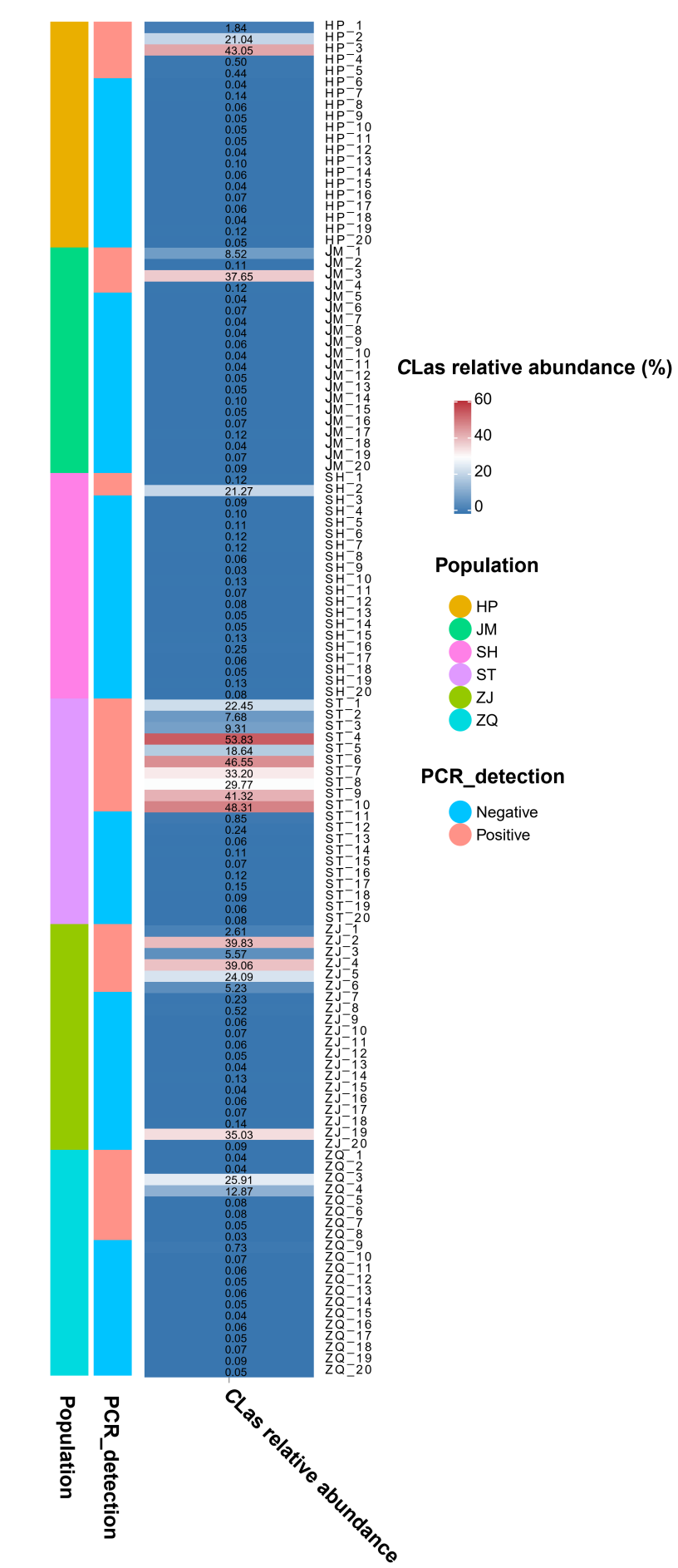


**Figure S1.** Heatmap showing the relative abundance of *C*Las in *D. citri* samples. Left color bar indicates the population to which the sample belongs. Right color bar indicates the conventional PCR detection result of the sample.

**
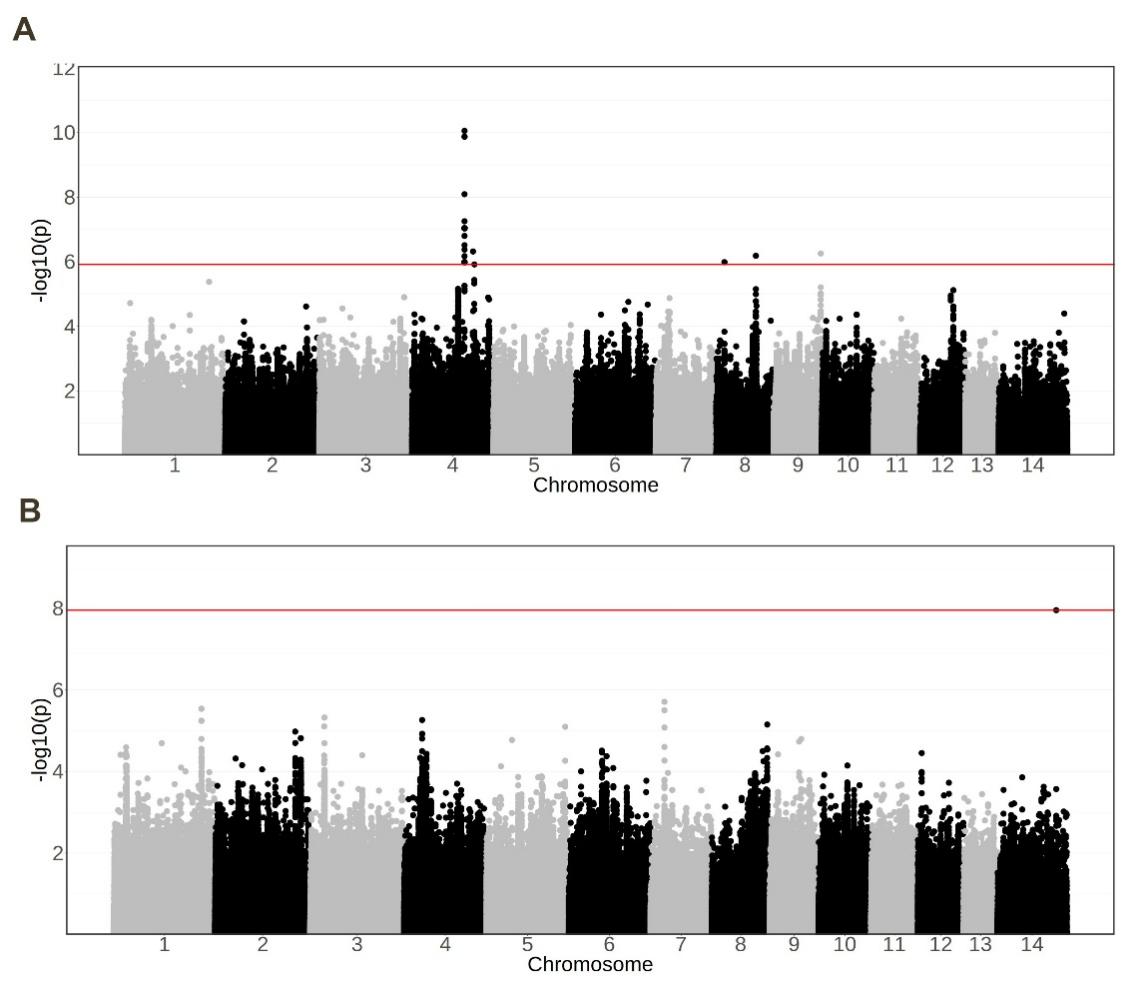
**

**Figure S2.** Manhattan plot of genome-wide associations between genetic variations and the relative abundance of *Candidatus* Profftella (A) and *Wolbachia* (B).


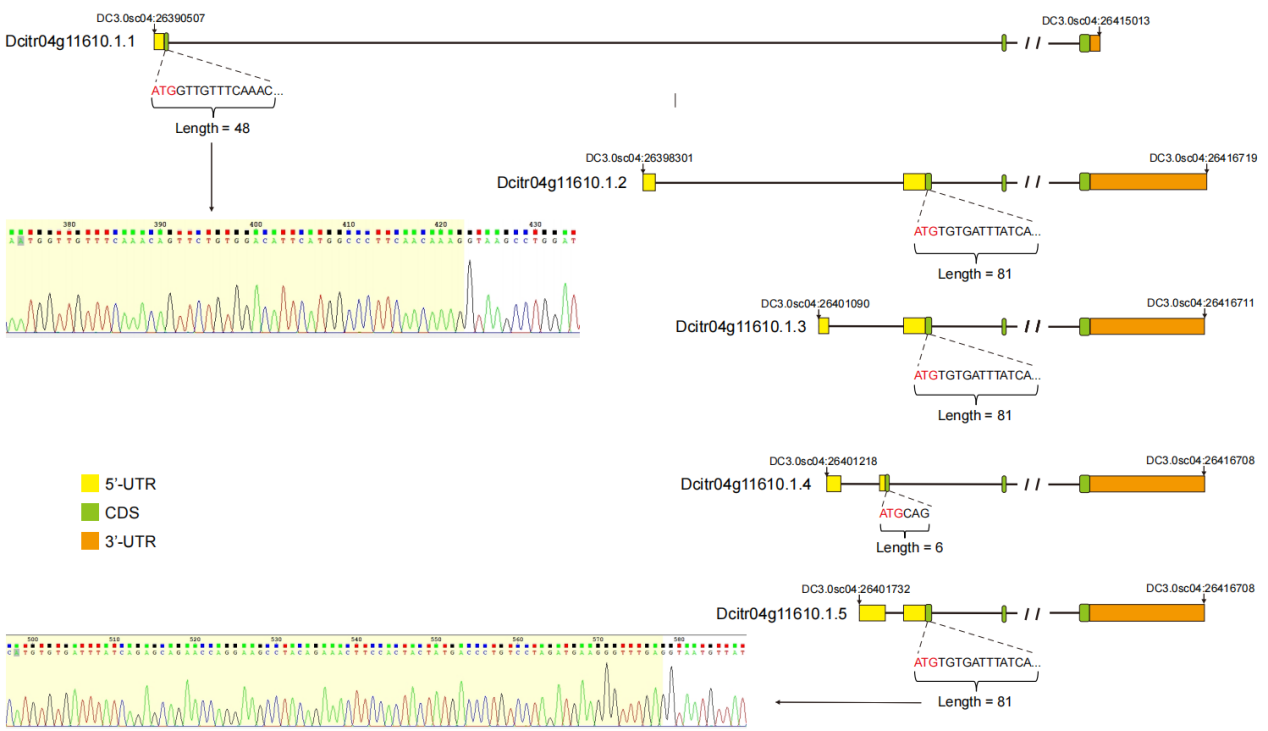


**Figure S3.** Genomic structure of gene *Dcitr04g11610.1*. Five transcripts of this gene are shown. Identical genomic regions for different transcripts are omitted in the figure. Yellow background in the sequencing chromatograph indicates the exon region.

**
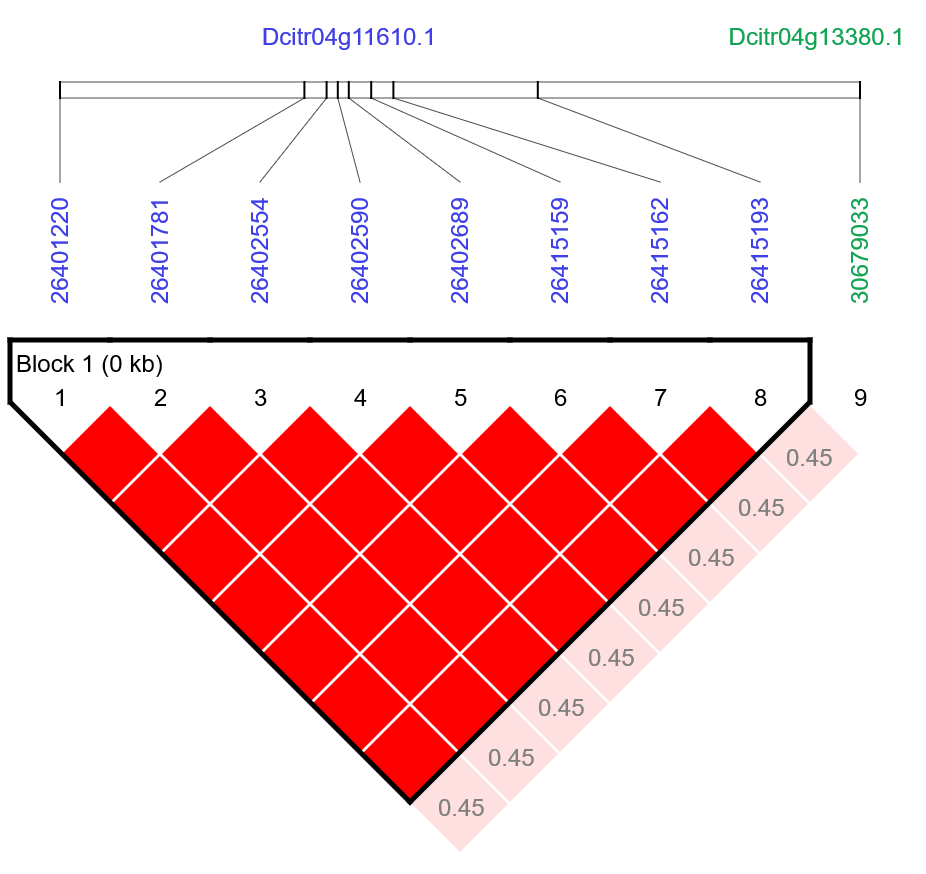
**

**Figure S4.** Linkage disequilibrium analysis of the significant single nucleotide polymorphisms in the gene loci of *Dcitr04g11610.1* and *Dcitr04g13380.1.* Number in grid represents the pairwise *D*’ values calculated using Haploview software. The red color in grid without number indicates a pairwise *D*’ value of 1.00 (complete linkage). A block structure was considered for marker pairs showing *D*’ > 0.80. This figure showed the SNP sites of gene *Dcitr04g11610.1* were complete linkage but had low linkage disequilibrium with the SNP of gene *Dcitr04g13380.1* within the same chromosome.


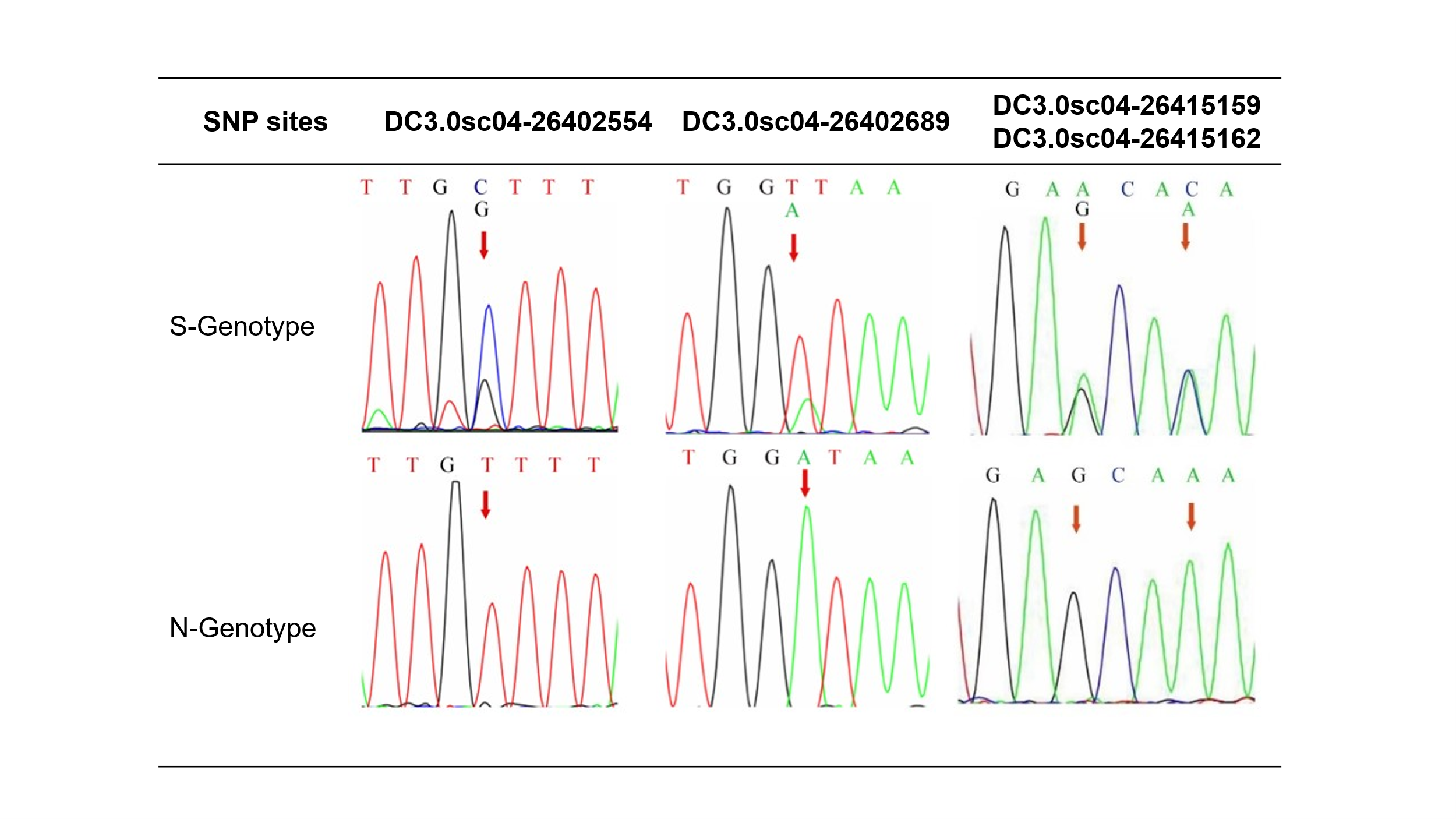


**Figure S5.** Sequencing chromatograph showing the genotypes of the significant single nucleotide polymorphisms in the gene loci of *Dcitr04g11610.1*. S-genotype: *C*Las-susceptible genotypes; N-genotype: normal genotypes. Note: Chromatographs were obtained by sequencing with reverse primers.

**
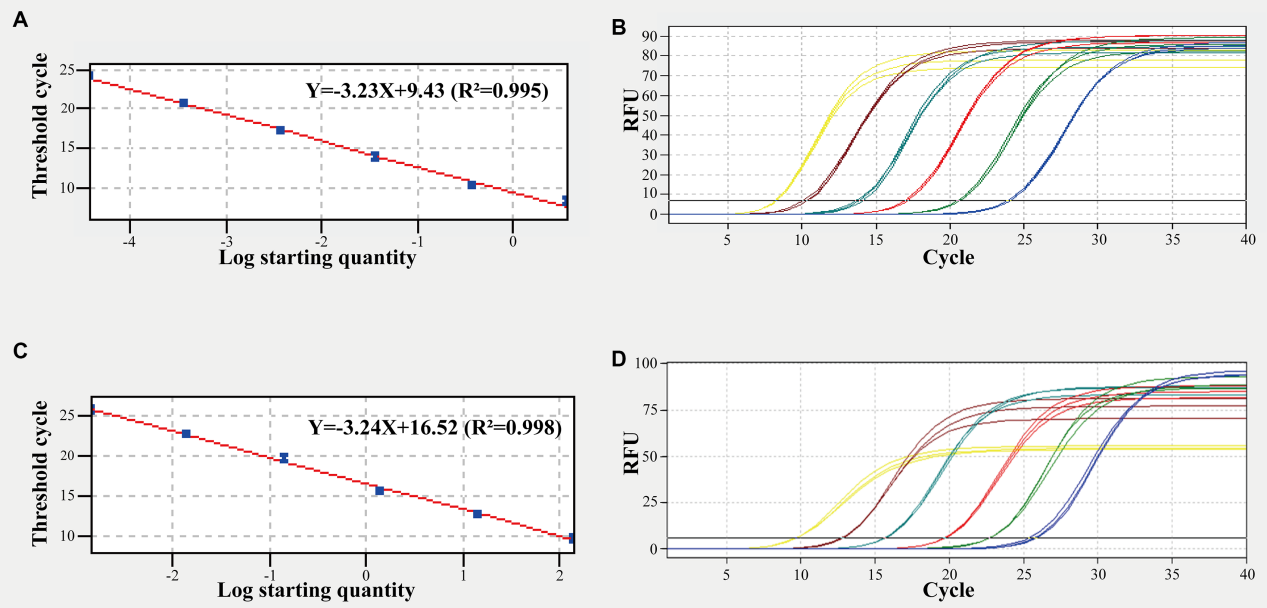
**

**Figure S6.** Standard curves and the corresponding equations of absolute quantification PCR detection for the abundance of *Candidatus* Liberibacter asiaticus (*C*Las) (A) and *Candidatus* Profftella armatura (C). The amplification efficiencies of the primers for *C*Las (B) and *Profftella* (D) are shown.
